# Effects of the Proactive Interdisciplinary Self-Management (PRISMA) Program on Online Care Platform Usage in Patients with Type 2 Diabetes in Primary Care: A Randomized Controlled Trial

**DOI:** 10.1155/2020/5013142

**Published:** 2020-01-08

**Authors:** Esther du Pon, Nanne Kleefstra, Frits Cleveringa, Ad van Dooren, Eibert R. Heerdink, Sandra van Dulmen

**Affiliations:** ^1^Research Group Process Innovations in Pharmaceutical Care, Utrecht University of Applied Sciences, PO Box 12011, 3501 AA Utrecht, Netherlands; ^2^Diabetes Centre, Isala, Zwolle, Netherlands; ^3^Medical Research Group, Langerhans, Ommen, 7731 MX, Netherlands; ^4^Department of GGZ Drenthe Research and High Intensive Care, GGZ Drenthe Mental Health Services, Assen, 9404 LA, Netherlands; ^5^Department of Internal Medicine, University of Groningen and University Medical Center Groningen, Groningen, 9713 GZ, Netherlands; ^6^Julius Center for Health Sciences and Primary Care, University Medical Center Utrecht, Utrecht, 3584 CX, Netherlands; ^7^Utrecht Institute for Pharmaceutical Sciences, Utrecht University, Utrecht, Netherlands; ^8^Nivel (Netherlands Institute for Health Services Research), Utrecht, 3513 CR, Netherlands; ^9^Department of Primary and Community Care, Radboud University Medical Center, Radboud Institute for Health Sciences, Nijmegen, 6525 GA, Netherlands; ^10^Faculty of Health and Social Sciences, University of South-Eastern Norway, Drammen 3045, Norway

## Abstract

Online care platforms can support patients with type 2 diabetes (T2DM) in managing their health. However, in the use of eHealth, a low participation rate is common. The Proactive Interdisciplinary Self-Management (PRISMA) program, aimed at improving patients' self-management skills, was expected to encourage patients to manage their disease through the use of an online platform. Therefore, the objective of the current study was to investigate whether a group education program can improve the use of an online care platform in patients with T2DM treated by primary care providers in the Netherlands. In a randomized controlled trial, patients with T2DM received either PRISMA with usual care or usual care only. During a six-month follow-up period in 2014-2015, usage (number of log-ons and time spent per session) of an online care platform (e-Vita) aimed at improving T2DM self-management was assessed. A training about the functionalities of e-Vita was offered. The sample consisted of 203 patients. No differences were found between the intervention and control groups in the number of patients who attended the platform training (interested patients) (*X*^2^(1) = 0.58; *p* = 0.45), and the number of patients who logged on at least once (platform users) (*X*^2^(1) = 0.46; *p* = 0.50). In addition, no differences were found between the groups in the type of users—patients who logged on twice or more (active users) or patients who logged on once (nonactive users) (*X*^2^(1) = 0.56; *p* = 0.45). The PRISMA program did not change platform usage in patients with T2DM. In addition, only a small proportion of the patients logged on twice or more. Patients probably need other encouragements to manage their condition using an online platform.

## 1. Introduction

Worldwide, the prevalence of diabetes mellitus is increasing dramatically. In the Netherlands, 66 per 1,000 persons have type 2 diabetes mellitus (T2DM), and this rate is expected to increase to 80 per 1,000 persons by 2025 [1]. Although patients with T2DM are primarily treated by primary care providers, this projected growth is expected to exceed the number of available providers [2]. Already, diabetes care providers see an increase in patients, which results in a decrease in face-to-face time available per patient. This has partly been tackled by a transfer of tasks, previously performed exclusively by general practitioners (GPs), to other medical professionals including specialized practice nurses (PNs). To deal with the increasing number of patients with T2DM and the burden of diabetes on healthcare, increased patient participation is needed, including more self-management. Self-management includes the active participation of patients in their treatment [3] to minimize the impact of chronic disease on their physical health and functioning and to enable patients to cope with the psychological effects of the illness [4]. Patient participation could be enhanced by offering them the possibility to track their own medical data together with tailored advice through eHealth [5].

eHealth applications, and more specifically online care platforms, provide the opportunity for self-management support and maintaining and/or improving the quality of chronic disease management by engaging patients in their own healthcare [6]. In general, online care platforms are environments in which patients can get an overview of their health outcomes, communicate with their care provider, and/or read information regarding their disease. It has been shown that such platforms are beneficial for people with T2DM [7]. These platforms have the potential to support patients in managing their own health and changing their lifestyle [8].

So far, the effects of online care platforms reported in systematic reviews vary [9–11]. Health behaviors and health-related outcomes have been shown to improve through the use of eHealth [12–15]. Moreover, these platforms were shown to be specifically beneficial for people with T2DM [7]. Therefore, platforms aimed at empowering patients can potentially decrease the workload of diabetes care providers and improve the (cost-) effectiveness of diabetes treatment [16, 17]. Nevertheless, implementation problems, nonadherence, and low participation are common [18–23]. A recent literature review of studies reporting online care platform use by patients with diabetes (type 1 and 2) revealed that 29% to 46% of them registered for a platform account; of those registered, 27% to 76% of patients used the platform at least once [23]. Platform use was associated with the following factors: patient characteristics (e.g., sociodemographic, clinical characteristics, and health literacy), technology (e.g., functionality, usability), and provider engagement. For facilitating the use of self-management support through a platform, patients first need to develop an intention for behavioral change, which can only be achieved if they have sufficient risk awareness, experience a need for behavioral change, and feel confident in making these changes [24]. In the Netherlands, an online care platform called e-Vita has been developed to improve patients' self-management skills [25].

Group education could be a helpful way for patients to obtain an intention for behavioral change [26]. A recent systematic review suggests that group-based diabetes self-management education is associated with improved clinical and psychosocial outcomes [27]. The Proactive Interdisciplinary Self-Management (PRISMA) program is aimed at improving self-management skills in patients with T2DM [26]. PRISMA appeared to improve self-management behavior in terms of dietary behaviors, foot care, action planning, and medication adherence [24, 28]. In addition, a pilot study showed that the PRISMA program is promising for decreasing dietary intake in newly diagnosed, overweight patients with T2DM in secondary care [26]. PRISMA helps patients to evaluate their own risk factors, to set personal goals, and to formulate a realistic action plan. Therefore, the PRISMA program is expected to increase the patients' motivation to behavior change and to motivate patients to manage their condition using an online platform.

The objective of the current study was to investigate whether a group education program aimed at empowering and stimulating self-management in patients with T2DM can improve the use of an online care platform.

## 2. Material and Methods

### 2.1. Study Design

**Figure 1:**
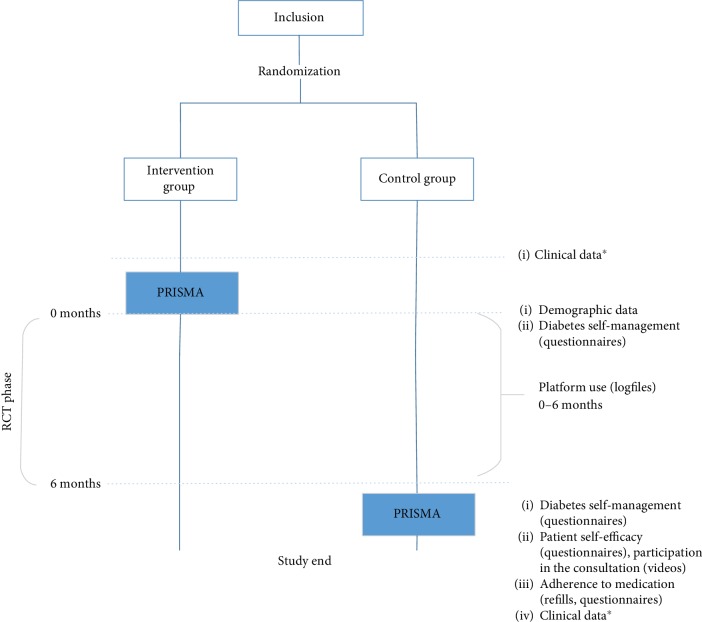
Overview of the complete DESTINE trial of which the current study is part of PRISMA = Proactive Interdisciplinary Self-Management; RCT = randomized controlled trial. ^∗^Clinical data includes HbA1c, body mass index (BMI), systolic blood pressure, and cholesterol levels.

The current study is part of the Diabetes Education and Self-Management to Increase Empowerment (DESTINE) study described in detail elsewhere [29]. DESTINE is a randomized controlled trial that followed enrolled patients for six months (Figure 1). The patients with T2DM received either the PRISMA program with usual care or usual care only. According to the guidelines of the Dutch College of GPs (NHG-Standard), usual care involves two to four visits per year with a PN and one annual check-up with a GP. All patients had access to the online care platform.

In another substudy of DESTINE, the effects of PRISMA on medication adherence were described [28]. Therefore, people 18 years old or older who diagnosed with T2DM and treated in primary care were included. Eight general practices in the eastern part of the Netherlands participated, and eligible patients were selected by GPs. Nonstratified block randomization was used to allocate participants to one of the two groups [30]. For the current study, we followed the methods of du Pon et al. [28].

### 2.2. Description of the Platform

In a previous study, the online care platform used for this study (e-Vita) showed that users of the platform had lower glycated hemoglobin (HbA1c) levels and reported higher quality of life, better well-being, lower diabetes-related distress, and better medication adherence than non-users [5]. However, the usage of this platform has been shown to be minimal in the past [5, 31]. Only 11% of the patients who were interested in the use of a care platform logged on at least once [31]. An improved version of the online platform e-Vita was used in our study. First, platform adjustments were conducted to make the platform more user-friendly. Second, the log-on procedure was simplified. Third, a function regarding communication between patients and their PN was added. Finally, a training was offered to patients, their companions, and caregivers about the functionalities of e-Vita, as suggested by Roelofsen et al. [5].

The use of e-Vita was offered as part of a larger program that aimed to study the effects of an online platform for various chronic illnesses (T2DM, chronic obstructive pulmonary disease, and chronic heart failure). The platform (accessible through app.e-vita.nl) not only contains information about the patient's health status but also offers participants more engaging options, such as formulating personal goals, participating in educational modules, exchanging messages between patient and care provider, or searching for information in the “library.” The language of the platform is Dutch.  Table 1 shows these items in more detail.

In both groups, participants (and their spouses) had the option to use the platform. Therefore, participants were registered on e-Vita and were invited for a 90-minute training about this platform. In groups of 5–10, patients were introduced to e-Vita and received log-on data. Spouses were also invited due to their important role in supporting the patient who wish to access and use the platform [32]. They also became familiarized with the content by completing several exercises on the platform (e.g., “enter your weight” or “formulate a health goal”). After this training, participants were able to start using the platform immediately. The PNs also received training about how to use e-Vita to be able to respond to their patients' messages, answer questions about the platform, and follow their patients' activity in the educational modules.

### 2.3. Description of the Intervention

The intervention consisted of two group meetings about T2DM guided by care providers. The PRISMA program has been described in detail in du Pon et al. [29]. During PRISMA, patients were encouraged to set personal goals and formulate a realistic action plan. They could enter these personal goals on e-Vita and pursue their goals using the platform.

### 2.4. Outcomes

The outcome of this study was the change between groups in the usage of the e-Vita platform (number of log-ons and time spent per session), as assessed by user log files.

#### 2.4.1. Usage of the e-Vita Platform

**Figure 2:**
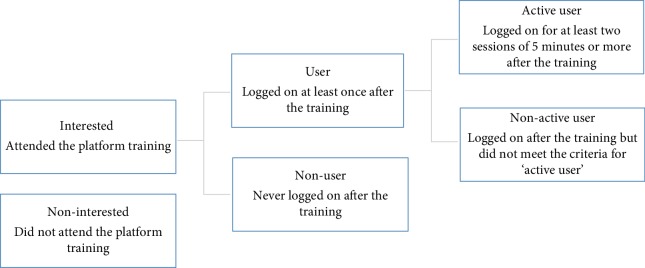
Flowchart of the process of sorting users into categories by log-on behavior.

Each action performed by the users was logged in a file that was saved to the server and was available for the researcher. Log-in and log-out times of each session of all platform users were registered. First, for all platform users, the number of log-ons, the time spent per session, and the total time spent on the platform were calculated. Then, patients were defined according to what type of platform user they were (Figure 2) [25]. A session included all log-ons to the platform within 30 minutes [33].

#### 2.4.2. Baseline Demographic and Clinical Data

Demographic baseline data were obtained from questionnaires, including sex, age, education level, health-related quality of life, emotional well-being, quality of received care, and eHealth literacy. Participants of the intervention group received the paper-based questionnaires immediately after the PRISMA program, whereas those of the control group received them by post.

Health-related quality of life was assessed by the EuroQol Five Dimension (EQ-5D-3L) scale [34]. This questionnaire consists of two parts: the EQ-5D descriptive system and the EQ visual analog scale (EQ-VAS). The EQ-5D-3L comprises five dimensions: mobility, self-care, usual activities, pain/discomfort, and anxiety/depression. Each dimension has three levels: no problems, some problems, and extreme problems. The EQ-5D-3L includes a visual analog scale (EQ-VAS) from which an individual rates their own health today from 0 (worst imaginable health) to 100 (best imaginable health). Emotional well-being was assessed by the 5-Item World Health Organization Well-Being Index (WHO-5) scale [35]. The WHO-5 covers five items: subjective quality of life based on positive mood (good spirits, relaxation), vitality (being active, waking up fresh and rested), and general interest (being interested in things). Each of the five items is rated on a 6-point Likert scale from 0 (not present) to 5 (constantly present). Higher scores represent higher levels of emotional well-being. Quality of received care was assessed by the HowRwe [36]. This questionnaire has four items concerning promptness, communication, personal relationship, and general satisfaction. Each item is scored using four levels ranging from “excellent” to “poor,” and each level is assigned a score on a scale from 0 (poor) to 3 (excellent), with higher scores indicating a better patient experience.

eHealth literacy was assessed by the eHEALS questionnaire, an 8-item scale measuring perceived skills at finding, evaluating, and applying electronic health information to health problems [37]. For this study, the four most relevant items were used (*α* = .90): (1) “I know how to find helpful health resources on the Internet”; (2) “I know how to use the Internet to answer my health questions”; (3) “I know what health resources are available on the Internet”, and (4) “I feel confident in using information from the Internet to make health decisions”. The items were measured with a 5-point Likert scale with response options ranging from “strongly disagree” to “strongly agree.” Total scores of the eHEALS were summed (possible range, 4–20), with higher scores representing higher self-perceived eHealth literacy.

In addition, baseline clinical data (T2DM duration, HbA1c, body mass index, and systolic blood pressure) were obtained from the personal health record systems of the GPs. Only data gathered less than four months before the intervention were used.

### 2.5. Sample Size Calculation

To show a difference of at least 20% in active users (15% in the control group versus 35% in the intervention group) for the primary outcome measure (usage of the e-Vita platform) with a two-sided risk alpha of 5% and a power of 80%, 81 individuals per group were needed using the unpooled *Z*-test. With an expected drop-out rate of 20%, we aimed to include 200 patients in our randomized controlled trial.

### 2.6. Analysis

Log files were collected for each patient over a six-month period. An intention to treat (ITT) analysis and a per protocol (PP) analysis were conducted. The PP analysis consisted of all patients of the intervention group who attended at least one session of the PRISMA program.

### 2.7. Statistical Analysis

All analyses were conducted using IBM SPSS Statistics version 22. Normally distributed data were presented as the means and standard deviation, whereas skewed data were presented as the medians and interquartile range. Dichotomous/categorical data were presented as numbers and percentage of the total. To evaluate differences in target variables (use of the online platform: number of log-ons and time spent per session) over time and between arms, the chi-square test and median difference scores (95% confidence intervals [CI]) were used. A subgroup analysis was conducted to investigate differences in platform use between male and female patients, younger (<65 years) and older patients (>65 years), and patients with a low eHEALS sum score (12 or less) and patients with a high eHEALS sum score (13 or more).

### 2.8. Ethics

This study was reviewed by the Medical Ethics Committee of Isala, Zwolle, the Netherlands. The committee decided that formal approval was not necessary (METC no. 14.07104).

## 3. Results

**Figure 3:**
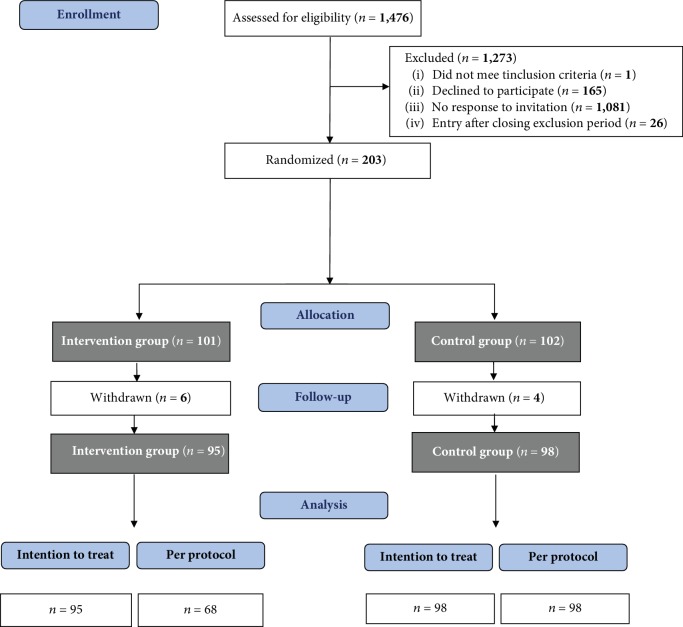
Flow chart of the participant selection process. Participants in the per protocol analysis attended at least one session of the PRISMA program.

The inclusion lasted nine months (June 2014 to February 2015). Of 1,476 eligible patients, 203 (13.8%) were included in the study and signed the informed consent form; 101 patients were randomized to the intervention group and 102 were randomized to the control group. After inclusion, 10 patients (4.9%) withdrew from the study: 6 in the intervention group and 4 in the control group. Patients withdrew because of illness, immigration, or personal reasons. In the intervention group, 68 (71.6%) of 95 patients attended at least one of the two PRISMA meetings. The CONSORT patient flow chart is presented in Figure 3 [38].

### 3.1. Patient Characteristics

The patient characteristics are presented in Table 2. Of the total sample (*n* = 193), 60.1% were men. The mean age was 69.9 years (SD, 9.1; range, 35–96). No differences in patient characteristics were found between groups at baseline.

### 3.2. Platform Usage

The results of the ITT analyses on the platform usage are presented in Table 3.

Of the 193 participants in total, 58 (61.1%) patients of the intervention group and 65 (66.3%) patients of the control group registered on the platform during the training (*interested patients*). At 6 months, 33 (34.7%) patients of the intervention group and 33 (33.7%) patients of the control group had logged on to e-Vita once or more (*platform users*). Of the intervention group, 12 (12.6%) patients logged on once (*nonactive users*); 15 (15.3%) patients of the control group logged on once. Of the intervention group, 21 (22.1%) patients logged on twice or more (*active users*); 18 (18.4%) patients of the control group logged on twice or more. No differences were found between the groups in the number of interested patients (*X*^2^(1) = 0.58; *p* = 0.45), the number of platform users (*X*^2^(1) = 0.46; *p* = 0.50), or the type of users (*X*^2^(1) = 0.56; *p* = 0.45).

Males more often were *interested patient* compared to females (*X*^2^(1) = 7.70; *p* = 0.00). However, no association was found between sex and being a *platform user* (*X*^2^(1) = 1.41; *p* = 0.24) or being an *active user* of the platform (*X*^2^(1) = 0.83; *p* = 0.36). Also, no association was found between age and being an *interested patient* (*X*^2^(1) = 0.12; *p* = 0.73), a *platform user* (*X*^2^(1) = 0.26; *p* = 0.61), or *active user* of the platform (*X*^2^(1) = 0.10; *p* = 0.92). In addition, no association was found between self-perceived eHealth literacy and being an *interested patient* (*X*^2^(1) = 0.71; *p* = 0.39) or *active user* of the platform (*X*^2^(1) = 0.62; *p* = 0.43). However, patients with a higher self-perceived eHealth literacy more often were *platform users* than patients with a lower self-perceived eHealth literacy (*X*^2^(1) = 5.01; *p* = 0.03).

Of all patients, the median number of log-ons was 0 (interquartile range, 0–1) for the intervention group and 0 (interquartile range, 0–1) for the control group. Of the interested patients, the median number of log-ons was 2 (interquartile range, 1–7) for the intervention group and 2 (interquartile range, 1–5) for the control group with a median difference score of –0.34 (95% CI, –3.28 to 2.61). The total time spent on the platform was 56 minutes (interquartile range, 00 : 27–02 : 15) for the intervention group and 53 minutes (interquartile range, 00 : 27–02 : 14) for the control group with a median difference score of 0.90 (95% CI, –50.25 to 52.05).

The results of the PP analysis do not differ from the ITT results above (data not shown).

## 4. Discussion

The PRISMA group program, which aimed to improve T2DM patients' self-management skills, was expected to increase patient motivation for behavior change and encourage them to manage their disease through the use of an online platform. However, PRISMA did not result in a higher platform usage of patients with T2DM over a six-month period. No differences were found between the groups regarding the number of log-ons, the length of time spent per session, and the total time spent on the platform. In addition, attending the PRISMA intervention did not increase the chance of becoming a user of the platform or enhanced patient activity on the platform. Clearly, consideration of their own personal risk factors and choice of a specific behavior change goal during PRISMA did not encourage patients (enough) to actually use the platform.

Previous results on the effect of e-Vita usage on clinical outcomes were promising [25]. However, our study showed that usage of this platform was minimal. Even offering the PRISMA program coupled with a training to introduce the platform did not remove the threshold for patients to use the platform. Most of the patients who were registered for platform use and attended the training never logged on. Going online indeed requires extra effort for which the added value in view of self-managing a chronic disease using eHealth may not be automatically apparent for patients [39]. An explanation could be either a lack of worry about their future health [40, 41], resulting in an insufficient intrinsic motivation, and no intention to change behaviors. In addition, the absence of disease burden in early-stage T2DM could lead to a lack of patient motivation to use an online health platform. Most complications of T2DM arise on longer term. It would be easier to motivate patients if they could already see results of their self-management behavior in the short term. In addition, the mean age of the patient group was 70 years, and we did not find an association between age and platform use; however, according to another study, younger age is associated with platform use [23]. Although the Netherlands is a country with high levels of general Internet diffusion even among older adults [42], these new techniques may be not suitable for older generations who have not mastered the required computer skills [43]. We showed that male patients more often were interested in platform use compared to female patients, which corresponds to the findings of Roelofsen et al. [5]. Our finding that high eHealth literate patients used the platform more often than low eHealth literate patients corresponds to the literature [44, 45]. The improvement of eHealth literacy in patients with T2DM needs attention. A recent literature review revealed the lack of data accuracy as the most important barrier to using eHealth for patients with T2DM [45]. This was often a result of manually reporting or the input of monitoring data. In our study, we recognized this problem. Patients probably need other encouragement to manage their T2DM through a platform, for example by follow-up meetings, counsel sessions, or reminders. Otherwise, a platform might not be the most appropriate solution to improve self-management skills in patients with T2DM. The day-to-day management of T2DM can be complex and challenging and requires major responsibility of patients. The use of an online platform would be an extra activity and, as a result, patients could become overwhelmed. In addition, T2DM often affects people with a lower socioeconomic status [46], who could experience other problems considered more important than their chronic condition.

In our study, 34% of the patients logged on, which was an improvement from the 11% found in the study by Roelofsen et al. (2014) on the former version of e-Vita. The trainings about the functionalities of the platform seemed necessary to familiarize patients with the platform [45, 47, 48]. This may also have decreased the threshold for patients to visit this platform and may have helped patients and PNs to feel the gains of using it. According to Sieverink (2014), PNs understand the importance of stimulating self-management skills of patients with chronic conditions via a platform. Other possible causes for the increase in platform usage could be the visual and technical improvements of the platform. In addition, bended care, where digital health and usual care are integrated, is likely to lead to increased use of an online program for patients with COPD [48]. Patients with diabetes log on to platforms for various reasons. According to the literature, frequently used features are viewing laboratory results [44], sending and reading messages [49], ,and participating in educational modules [50]; setting personal goals is the least popular feature [50].

### 4.1. Strengths and Limitations

To our knowledge, this is the first study that focuses on the extent to which patients use an online diabetes care platform after attending a group education program. The training offered about the functionalities of e-Vita helped patients to start their first session on the platform. In addition, communication between healthcare providers and patients, a new function on e-Vita, may have encouraged the use of the platform. Another strength of the study was that the participants were randomized over all general practices. This prevented influences from the general practices on the results. In addition, the study has pragmatic character, which means that it was designed to test PRISMA in the full spectrum of everyday usual care to maximize applicability and generalizability. Therefore, the study determined whether PRISMA actually increased the platform usage in real life. In 2017, the platform was renewed, extended with an app, and used by twenty care groups in the Netherlands.

Some limitations of this study need to be mentioned as well. The eight participating general practices diverged by inclusion rate: of the 1,476 invited patients, most were registered in three general practices. In addition, despite the instructions given during the training, the steps to gain access to the platform were considered complex. These factors posed a considerable barrier to visiting the platform, especially for those who were less able to handle computers. In the beginning of the project, technical problems appeared on the platform and, as a result, outcomes of patient annual checkups were not displayed. Furthermore, despite our efforts to enthuse patients about the PRISMA program, only 12% of the approached patients participated. Our target group might not be interested in this type of intervention, which could explain this low participation rate. In addition, most participants in our study were moderately or highly educated and reported a moderate or high eHealth literacy. Lower educated patients, in whom T2DM is most common [51], may not be interested in interventions such as PRISMA and e-Vita because of its complexity. Despite the randomized design, whether a patient becomes a (active) user of e-Vita could thus depend on caregiver attitudes toward participation, online platforms, and eHealth in general (selection bias).

## 5. Conclusions

The PRISMA program did not result in a higher online healthcare platform usage in patients with T2DM, and also, the continuity of use was low. The added value of self-managing a chronic disease may not be automatically apparent for patients. In addition, platforms may not be suitable for the older adults with lower eHealth literacy. There is a need to invest in developing online skills, particularly for older adults with lower eHealth literacy. Otherwise, these patients probably need other encouragements to help them manage T2DM using an online platform. Future research should explore other sources for patients to develop intentions to behavior change for facilitating the use of self-management support programs within a platform. Further development of the platform is ongoing.

## Figures and Tables

**Table 1:** Platform items.

Health status	View annual checkups for the last three visits. Every outcome was accompanied by an explanation.
Personal goals	Formulate personal goals to reach health-related wishes.
Educational modules	Participate in education presented in text and pictures, followed by a set of simple control questions. This education was patient specific, based on their health data.
Messages	Exchange emails with PN through email program.
“Library”	Look at links to reliable information on T2DM in general, patient associations, and short videos about patient experiences using the platform.

**Table 2:** Patient characteristics at baseline (*n* = 193).

*n* (%)/mean ± SD/median (25-75 quartiles)	Intervention group (*n* = 95)	Control group (*n* = 98)
Male	56 (58.9)	60 (61.2)
Age in years	69.7 ± 9.8	70.1 ± 10.1
Education level^a^		
Low	4 (4.2)	8 (8.2)
Moderate	41 (43.2)	45 (45.9)
High	12 (12.6)	11 (11.2)
Unknown	38 (40.0)	34 (34.7)
T2DM duration (years)	6 (4–8)	6 (4–9)
HbA1c (mmol/Mol)	50.7 ± 8.5	54.7 ± 11.7
BMI	28 (26–31)	30 (27–34)
Systolic blood pressure (mmHg)	139 (131–150)	130 (126–148)
Cholesterol (mmol/L)	4.3 (3.5–5.1)	3.9 (3.4–4.9)
EQ-5D index score	0.9 (0.8–01)	0.9 (0.9–1.0)
WHO-5 index score	76 (61–80)	80 (72–80)
HowRwe sum score	12 (10–12)	12 (9–12)
eHealth literacy	13 (11–16)	13 (11–16)

^a^Low, no education or primary education; moderate, lower secondary education, (upper) secondary education, or post-secondary non-tertiary education (including vocational education); high, tertiary education (bachelor's degree or higher). T2DM = type 2 diabetes mellitus; HbA1c = glycated hemoglobin; BMI = body mass index; EQ-5D = EuroQol Five Dimension; WHO-5 = 5-Item World Health Organization Well-Being Index.

**Table 3:** Results of platform usage by interest, user category, and activity.

*n* (%)/mean ± SD/median (25-75 quartiles)	Intervention group (*n* = 95)	Control group (*n* = 98)	*p* value/median difference score (95% CI)
Interested patient^a^	58 (61.1)	65 (66.3)	0.45^∗^
Platform user^b^	33 (34.7)	33 (33.7)	0.50^∗^
Active user^c^	21 (22.1)	18 (18.4)	0.45^∗^
Number of log-ons (all patients)	0 (0–1)	0 (0–1)	0.77#
Number of log-ons (only interested patients)	2 (1–7)	2 (1–5)	0.74#
Range	1–11	1–26	−0.34 (−3.28 to 2.61)
Categories			0.85^∗^
0–1 log-ons	36 (63.2)	46 (70.8)	
2–4 log-ons	9 (15.8)	9 (13.8)	
More than 4 log-ons	12 (21.1)	10 (15.4)	
Time spent per session (hh/mm)	00 : 23 ± 00 : 09	00 : 25 ± 00 : 08	0.09#
Total time spent (hh/mm)	00 : 56 (00 : 27–02 : 15)	00 : 53 (00 : 27–02 : 14)	0.93#
Range	00 : 07–09 : 02	00 : 06–09 : 34	0.90 (–50.25 to 52.05)

^∗^Pearson chi-square test. #Mann–Whitney *U* test. ^a^Attended the platform training. ^b^Logged on at least once after the training. ^c^Logged on for at least two sessions of 5 minutes or more after the training.

## Data Availability

The datasets generated and/or analyzed during the current study are not publicly available because public access was not included in the informed consent.
